# Feasibility and safety of cavotricuspid isthmus ablation using exclusive intracardiac echocardiography guidance: a proof-of-concept, observational trial

**DOI:** 10.3389/fcvm.2023.1244137

**Published:** 2023-10-12

**Authors:** Dorottya Debreceni, Kristof-Ferenc Janosi, Marton Turcsan, Daniel Toth, Botond Bocz, Tamas Simor, Peter Kupo

**Affiliations:** Heart Institute, Medical School, University of Pecs, Pecs, Hungary

**Keywords:** atrial flutter, cavotricuspid isthmus ablation, intracardiac echocardiography, zero fluoroscopy, fluoroless ablation

## Abstract

**Introduction:**

Catheter ablation is the preferred treatment for typical atrial flutter (AFl), but it can be challenging due to anatomical abnormalities. The use of 3D electroanatomical mapping systems (EAMS) has reduced fluoroscopy exposure during AFl ablation. Intracardiac echocardiography (ICE) has also shown benefits in reducing radiation exposure during AFl ablation. However, there is a lack of evidence on the feasibility of ICE-guided, zero-fluoroscopy AFl ablation without the use of EAMS.

**Methods:**

In this prospective study, we enrolled 80 patients with CTI-dependent AFl. The first 40 patients underwent standard fluoroscopy + ICE-guided ablation (Standard ICE group), while the other 40 patients underwent zero-fluoroscopy ablation using only ICE (Zero ICE group). Procedure outcomes, including acute success, procedure time, fluoroscopy time, radiation dose, and complications, were compared between the groups.

**Results:**

The acute success rate was 100% in both groups. Out of the 40 cases, the zero-fluoroscopy strategy was successfully implemented in 39 cases (97.5%) in the Zero ICE group. There were no significant differences in procedure time [55.5 (46.5; 66.8) min vs. 51.5 (44.0; 65.5), *p* = 0.50] and puncture to first ablation time [18 (13.5; 23) min vs. 19 (15; 23.5) min, *p* = 0.50] between the groups. The Zero ICE group had significantly lower fluoroscopy time [57 (36.3; 90) sec vs. 0 (0; 0) sec, *p* < 0.001] and dose [3.17 (2.27; 5.63) mGy vs. 0 (0; 0) mGy, *p* < 0.001] compared to the Standard ICE group. Total ablation time was longer in the Standard ICE group [597 (447; 908) sec vs. 430 (260; 750), *p* = 0.02], but total ablation energy [22,458 (14,836; 31,116) Ws vs. 17,043 (10,533; 29,302) Ws, *p* = 0.10] did not differ significantly. First-pass bidirectional conduction block of the CTI and acute reconnection rates were similar between the groups. No complications or recurrences were observed during the follow-up period.

**Conclusion:**

Our study suggests that zero-fluoroscopy CTI ablation guided solely by ICE for AFl is feasible and safe. Further investigation is warranted for broader validation.

## Introduction

Typical atrial flutter (AFl) is a common type of supraventricular tachycardia characterized by a macro-reentrant circuit that involves the cavotricuspid isthmus (CTI), a narrow strip of tissue connecting the inferior vena cava and the tricuspid valve (1). Catheter ablation is the preferred initial treatment for recurrent, symptomatic CTI-dependent AFl. This procedure creates a bidirectional conduction block across the CTI and has a high success rate both in the short term and long term, with a low complication rate (2). Although catheter ablation of the CTI has a high success rate (2, 3), it can be particularly challenging in some cases, often due to anatomical abnormalities (4–6).

Traditionally, electrophysiology procedures are commonly performed with fluoroscopy guidance, which exposes both patients and medical staff to potentially hazardous levels of ionizing radiation (7, 8).

The utilization of 3D electroanatomical mapping systems (EAMS) during ablation procedures for typical flutter has resulted in a significant reduction in exposure to fluoroscopy (4, 9, 10). Recent researches have demonstrated that it is feasible to perform fluoroscopy-free ablation procedures for typical flutter with the guidance of EAMS (5, 6, 11).

**Figure 1:**
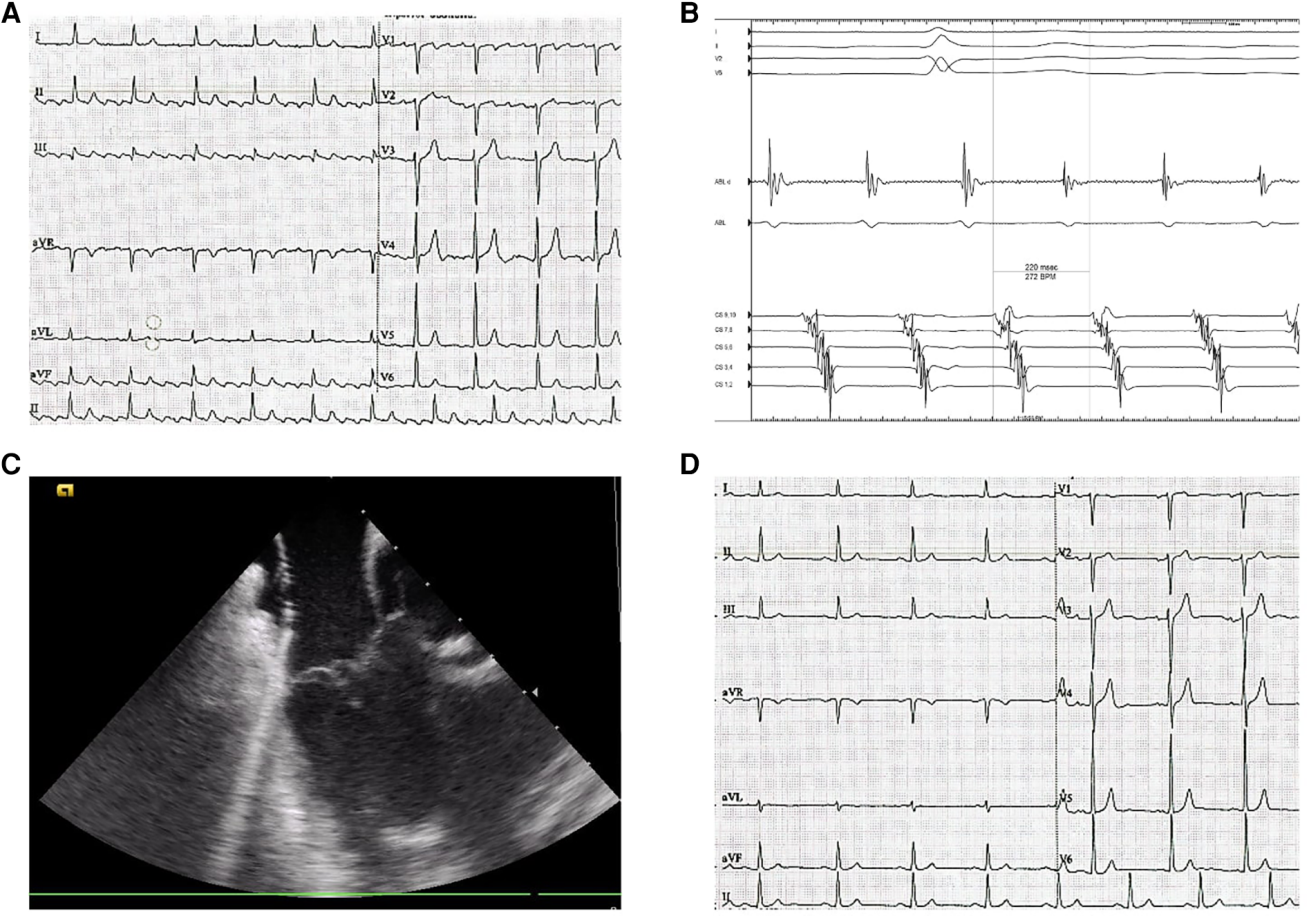
Typical flutter ablation. (**A**) preprocedural surface ECG (paper speed is 25 mm/s) displays the ongoing CTI-dependent flutter saw-tooth pattern. (**B**) coronary sinus (CS) electrocardiogram of a typical atrial flutter. (**C**) intracardiac echocardiography-guided cavotricuspid isthmus ablation. (**D**) postprocedural ECG (paper speed is 25 mm/s) demonstrates normal sinus rhythm.

**Figure 2:**
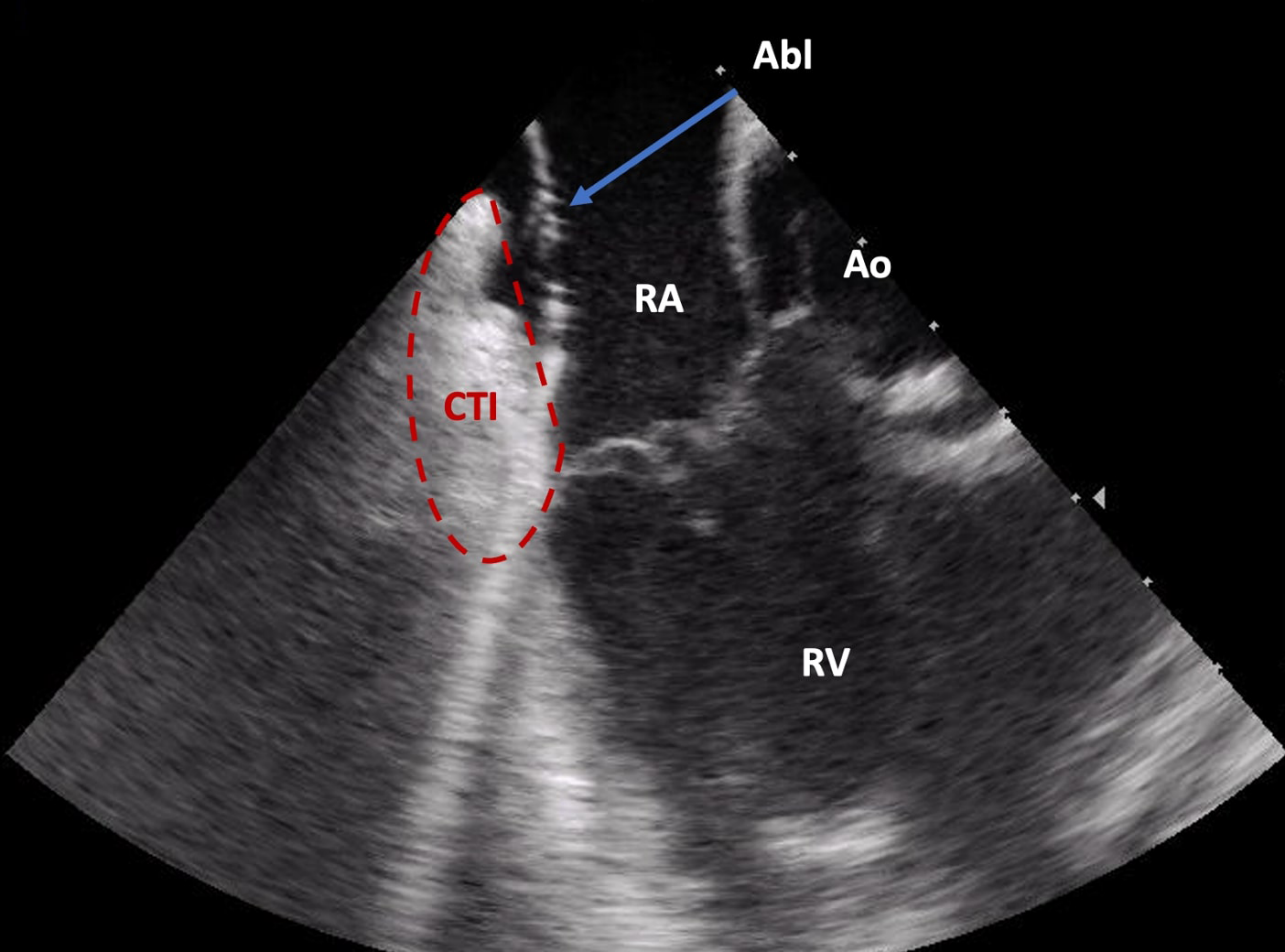
Direct visualization of the ablation catheter on the cavotricuspid isthmus by intracardiac echocardiography (ICE). Abl, ablation catheter; Ao, aortic root; CTI, cavotricuspid isthmus; RA, right atrium; RV, right ventricle.

Intracardiac echocardiography (ICE) is a distinctive imaging modality that provides real-time visualization of intracardiac structures during catheter ablation procedures. A prior clinical trial demonstrated that ICE-guided ablation of the CTI significantly reduces the duration of the procedure and fluoroscopy time, resulting in markedly decreased radiation exposure and less time required for ablation when compared to fluoroscopy-only procedures (Figures 1, 2) (4).

There is currently no scientific evidence available on the feasibility of ICE-guided, zero-fluoroscopy CTI ablation without the use of EAMSs. The primary goal of this prospective study was to assess the feasibility and safety of using solely ICE-guided, zero-fluoroscopy ablation techniques for ablation of the CTI in patients diagnosed with typical AFl. Additionally, the study aimed to compare the outcomes of this approach with the standard CTI ablation technique.

## Methods

### Study population

In our prospective, observational single-center study, we enrolled a total of 80 consecutive patients with ECG recordings suggesting ongoing or recent CTI-dependent AFl, based on ECG criteria as adopted by Granada et al. (1). The first 40 patients underwent standard ICE-guided CTI ablation according to our institutional protocol (Standard ICE group), while the other 40 patients underwent zero-fluoroscopy CTI ablation, using only ICE and intracardiac electrocardiograms for catheter navigation (Zero ICE group).

We excluded patients from the study if they met any of the following criteria: (a) being referred for a second procedure, (b) having other arrhythmias in addition to AFl, (c) having undergone cardiac surgery in the last 6 months, (d) having undergone previous pulmonary vein isolation, or (e) being under 18 years old. The study was conducted in accordance with the Declaration of Helsinki and was approved by the regional ethics committee. All patients provided written informed consent to participate in the study.

### Study protocol

Patients were assessed while under sedation (midazolam 2–5 mg administered intravenously) and/or analgesia (fentanyl 0.002–0.02 mg/kg) as needed. Three catheters were introduced after local anesthesia through the right femoral vein for the purposes of pacing, recording, and ablation. A decapolar steerable catheter (ViaCath 10, Biotronik, Germany) was positioned in the coronary sinus (CS), while an ablation catheter (Biotronik AlCath LT G FullCircle, Biotronik, Germany) and an additional 8F ICE catheter (AcuNaV ^TM^ 90 cm, Siemens Medical Solutions, Mountain View, CA, USA) was inserted into the right atrium. In the Standard ICE group, catheter placement was initially guided by fluoroscopy, whereas in the Zero ICE group, catheter manipulation was guided by ICE. No EAMS was used in either group. CTI dependent AFl was confirmed by entrainment maneuver in all patients with ongoing AFl. Radiofrequency ablation (RFA) was conducted by creating a linear lesion at the middle of the CTI from the tricuspid annulus to the inferior vena cava (Supplementary Video S1). RFA was performed in a temperature-controlled mode, targeting a temperature of 43°C, with power limited to 35–45 W and an irrigation rate of 15–30 ml/min. After the arrhythmia was terminated as a result of RFA, the procedure was concluded with CS stimulation. In patients exhibiting sinus rhythm, mapping and ablation were carried out while applying CS stimulation with a cycle length of 600 msec. All the procedures were conducted by an experienced electrophysiologist who performs over 60 CTI ablations annually using ICE guidance.

### ICE-guided tracking and navigation

The ICE catheter was initially positioned within the right atrium, precisely at the 6 o'clock orientation. Subsequently, the CS catheter was advanced, and the presence of intracardiac signals indicating its arrival in the right atrium was registered. This event was visualized using the ICE probe with either clockwise or counterclockwise rotation. The CS catheter was then bent over through the tricuspid annulus while considering its position by ICE. A slight clockwise rotation of the CS catheter towards the CS ostium was performed, allowing visualization through a corresponding slight clockwise rotation of the ICE transducer from the home view. With gentle retraction of the CS catheter, ICE was used to confirm its entry into the CS ostium. Subsequently, the CS catheter was straightened and advanced within the CS with meticulous and deliberate movements, all of which were monitored using ICE. Once the CS catheter was appropriately positioned within the CS, an ablation catheter was introduced into the right atrium. Throughout the ablation procedure, continuous verification of the ablation catheter's location along the CTI was maintained. The objective was to create a continuous ablation line within the central portion of the CTI, commencing from the tricuspid valve and extending towards the inferior vena cava (IVC). Standard home view was employed to visualize the middle of the CTI. A slight counterclockwise rotation of the ICE transducer revealed the lateral aspect of the CTI, while a slight clockwise rotation enabled visualization of the septal portion of the CTI.

### Study endpoints

Acute success was defined as the presence of bidirectional conduction block along the isthmus by pacing from proximal CS and inferior lateral wall, characterized by widely spaced double potentials, which persisted for a duration of 20 min following the last RF ablation. First-pass block was defined as the occurrence of bidirectional conduction block of the CTI, either before or upon completion of the CTI line ablation, without the requirement of additional ablations. Procedure time, measured in minutes, was determined from the initial femoral puncture until the withdrawal of catheters. Fluoroscopy time and radiation dose were automatically recorded by the fluoroscopy system. Ablation data, including the total amount of delivered radiofrequency energy (expressed in Ws) and the duration of ablation (expressed in seconds), were calculated and stored by the EP recording system (CardioLab, GE Healthcare).

### Statistical analysis

The distribution pattern of the data was evaluated using Kolmogorov-Smirnov tests. All tests were conducted as two-tailed tests with a significance level of *p* < 0.05. Continuous data were expressed as either mean ± standard deviation (SD) or median (interquartile range, IQR), depending on appropriateness. Categorical variables were presented as absolute numbers and percentages. Chi-square test, *T*-test, and Mann–Whitney *U*-test were employed for appropriate comparisons. Statistical analyses were performed using SPSS 28 software (SPSS, Inc., Chicago, IL, USA).

## Results

In this study, we enrolled a total of 80 participants, with 40 assigned to the Standard ICE group and 40 to the Zero ICE group. The baseline characteristics, including gender distribution (female: 70.0% vs. 67.5%, *p* = 0.81) and age (49.6 ± 14.9 vs. 53.0 ± 13.4 years, *p* = 0.15), show no significant differences between the two groups (Table 1). All 80 cases successfully completed the procedural endpoint, resulting in a 100% acute success rate.

**Table 1:** Clinical characteristics of the study population.

	Standard ICE group (*n* = 40)	Zero ICE group (*n* = 40)	*p* value
Age (years)	49.6 ± 14.9	53.0 ± 13.4	0.15
Male (%)	28 (70)	27 (67.5)	0.81
Hypertension (%)	30 (75)	35 (87.5)	0.15
Diabetes mellitus (%)	14 (35)	18 (45)	0.36
Heart failure (%)	13 (32.5)	8 (20)	0.20
Coronary artery disease (%)	9 (22.5)	12 (30)	0.44
Chronic kidney disease (%)	0 (0)	3 (7.5)	NA
Prior stroke/TIA (%)	6 (15)	3 (7.5)	0.32
Atrial fibrillation (%)	5 (12.5)	10 (25)	0.15
COPD (%)	2 (5)	1 (2.5)	0.56
LA diameter (mm)	59.0 ± 9.0	58.9 ± 7.9	NA
Ongoing typical AFL (%)	23 (57.5)	21 (52.5)	0.65

AFL, atrial flutter; COPD, chronic obstructive pulmonary disease; ICE, intracardiac echocardiography; LA, left atrium; NA, not available; TIA, transient ischemic attack.

Ongoing typical AFl was detected in 57.5% of cases in the Standard ICE group and 52.5% of cases in Zero ICE group. Analysis of the data revealed no significant difference in procedure time [55.5 (46.5; 66.8) min vs. 51.5 (44.0; 65.5), *p* = 0.50] and puncture to first ablation time [18 (13.5; 23) min vs. 19 (15; 23.5) min, *p* = 0.50] between two groups. Additionally, the time from the first to the last ablation [16 (10; 31) min vs. 12 (5; 25.5) min, *p* = 0.16] and the time from puncture to the last ablation [35 (27; 50) min vs. 32 (24; 46.5) min, *p* = 0.51] also demonstrated similar results.

In the Zero ICE group, 39 out of 40 patients (97.5%) underwent fluoroless ablation, resulting in significantly lower fluoroscopy time [57 (36.3; 90) sec vs. 0 (0; 0) sec, *p* < 0.001]. The range in the Standard ICE group was 29–184 s, while in the Zero ICE group, it was 0–78 s. Similarly, the dose was also reduced [3.17 (2.27; 5.63) mGy vs. 0 (0; 0) mGy, *p* < 0.001] in comparison to the Standard ICE group.

In one case, fluoroless catheter insertion was unsuccessful due to the kinking venous system, but no fluoroscopy was used after the catheters reached the heart.

The total ablation time was longer in the Standard ICE group [597 (447; 908) sec vs. 430 (260; 750), *p* = 0.02], while total ablation energy did not differ significantly between the group [22,458 (14,836; 31,116) Ws vs. 17,043 (10,533; 29,302) Ws, *p* = 0.10]. First-pass bidirectional conduction block of CTI was achieved in 55% of cases in both groups, (22/40 patients vs. 22/40 patients, *p* = 1.0), and acute reconnections occurred at similar rates (25.3% vs. 35%, *p* = 0.31). However, all instances of acute reconnections were effectively eliminated through additional RF applications.

Throughout the study no complications were observed. Result are summarized in Table 2.

**Table 2:** Procedural parameters in the study population.

	Standard ICE group (*n* = 40)	Zero ICE group (*n* = 40)	*p* value
Total procedure time (min)	55.5 (46.5; 66.8)	51.5 (44.0; 65.5)	0.50
From puncture to first ablation (min)	18 (13.5; 23)	19 (15; 23.5)	0.50
Total ablation time (s)	597 (447; 908)	430 (260; 750)	0.02
Total ablation energy (Ws)	22,458 (14,836; 31,116)	17,043 (10,533; 29,302)	0.10
From first to last ablation time (min)	16 (10; 31)	12 (5; 25.5)	0.16
From puncture to last ablation time (min)	35 (27; 50)	32 (24; 46.5)	0.51
Total fluroscopy time (sec)	57 (36.3; 90) range: 29–184	0 (0; 0) range: 0–78	<0.001
Total fluoroscopy dose (mGy)	3.17 (2.27; 5.63)	0 (0; 0)	<0.001
First pass block (%)	55.0	55.0	1.0
Acute reconnection (%)	25.3	35.0	0.31
Acute success (%)	100	100	1.0
Complication (%)	0	0	NA

ICE, intracardiac echocardiography; NA: not available.

## Discussion

Our single-center, prospective observational study demonstrates that ICE-guided, zero-fluoroscopy CTI ablation is feasible and safe without influencing the procedural time, first-pass block and acute reconnection rates. The Zero ICE group demonstrated a shorter total ablation time compared to the other group. However, no significant difference was observed in total ablation energy, indicating that, on average, higher power (W) values were utilized for CTI ablation using the zero strategy. The zero-fluoroscopy strategy was unsuccessful in only one case during catheter placement, but no fluoroscopy was employed after the catheters were successfully positioned within the heart.

Catheter ablation is the preferred method for maintaining sinus rhythm in the chronic treatment of AFl and is more effective than amiodarone (2). Ablation of the CTI with a confirmed bidirectional conduction block results in a recurrence rate of less than 10% (2). However, CTI ablation can be challenging in certain cases due to anatomical factors such as long isthmus, prominent Eustachian ridges, and pouches. Various visualization methods, including right atrium angiography, transesophageal echocardiography, and ICE, have been studied to identify the association between deep pouch-like recesses, long isthmuses, prominent Eustachian ridges, and increased procedure time, radiation exposure, and required RF ablations during CTI ablation for typical AFl (9, 10, 12).

Extended exposure to radiation can heighten the risk of dermatitis, cataracts, congenital anomalies, and elevate the likelihood of developing cancer in individuals who have been exposed (13). Additionally, the use of lead aprons is linked to an increased incidence of work-related musculoskeletal pain (14). Minimizing the utilization of fluoroscopy, or ideally eliminating it entirely, is of utmost importance due to the potential harm even from low doses and the absence of a clearly defined threshold for completely safe radiation exposure. Therefore, during electrophysiology procedures, the aim is to keep the use of ionizing radiation as low as reasonably achievable (ALARA principle).

In recent years, the use of EAMSs as an alternative visualization technique in electrophysiology procedures has gained popularity due to its potential to decrease procedural time and reduce or even completely eliminate radiation exposure. Numerous studies have confirmed the feasibility of near-zero and zero-fluoroscopy approaches for CTI ablation, including their successful application as an extension to pulmonary vein isolation procedures within a single session (5, 15–23). In addition, the advent of visualizable steerable sheaths has yielded significant advancements in mitigating radiation exposure during catheter ablation procedures (24).

However, EAMSs have limited ability to directly visualize intracardiac structures, which makes them less advantageous in catheter ablation procedures for patients with atypical cardiac anatomy.

ICE is a real-time imaging modality that facilitates catheter ablations by providing visualization of intracardiac structures, catheter position and stability, as well as lesion formation (4, 10).

The application of ICE in cardiac arrhythmia ablation has been shown to be associated with significantly reduced fluoroscopy time, fluoroscopy dose, and procedure duration compared to ablation procedures conducted without the use of ICE (4, 25, 26).

In a previous study conducted by Bencsik et al., a randomized trial was carried out to compare conventional CTI ablations with ICE-guided CTI ablations in 102 patients (4). The findings of this study demonstrated that ICE-guided CTI ablation significantly reduces the duration of the procedure, fluoroscopy exposure, and ablation time, as well as the total amount of delivered RF energy, in comparison to the fluoroscopy-only guided method without the assistance of EAMS. In the fluoroscopy-only guided group, the option of additional ICE usage was available in cases where the ablation or fluoroscopy time was prolonged, leading to a crossover group. Among these patients, a total of 7 individuals switched to ICE-guided EPS from the fluoroscopic approach. Notably, in 6 out of the 7 cases, a pouch near the Eustachian-valve/ridge was observed, and shortly after the crossover, bidirectional isthmus block was achieved. The authors concluded that the variable anatomy of CTI is a primary factor contributing to unsuccessful procedures. By employing ICE, the target site for ablation can be visualized in real-time, allowing for the identification of anatomical variations that may influence catheter ablation of CTI. As a result, the utilization of ICE could be associated with reduced overall procedure time, fluoroscopy exposure, and ablation duration.

Herman et al. conducted a comparative study involving 79 patients who underwent CTI ablation for typical AFl using either an ICE-guided or fluoroscopy-only guided method (27). In line with Bencsik et al., authors reported reduced fluoroscopy time with association of the usage of ICE, moreover additional ICE approach in unsuccessful fluoroscopy-guided CTI ablation contributed a completed bidirectional line achievement. The use of ICE resulted in a longer total procedure time compared to not using it. The authors attribute this finding to the fact that the ICE group involved an additional vein puncture, which contributed to the overall duration of the procedure. Notably, the total procedure time was measured until successful hemostasis, hence the increase in procedure duration was primarily due to the additional vein puncture and the time required for proper hemostasis. Importantly, despite the extra 11F vein puncture in patients receiving anticoagulation, no major vascular complications were observed in the ICE group.

To the best of our knowledge, there is currently no scientific evidence available that specifically focuses on zero-fluoroscopy AFl ablation solely guided by ICE. However, a conference abstract presented findings on 27 CTI ablation procedures where a zero-fluoroscopy approach was safely achieved using ICE without EAMS (28). All procedures could be successfully accomplished without the need for fluoroscopy. In one patient, an asymptomatic intramural hematoma was detected by ICE following the successful ablation, while no other major or minor complications were observed or documented throughout the study.

Compared to EAMSs, the utilization of ICE allows for real-time catheter navigation within the heart. This approach can lead to a more rapid and efficient procedure while effectively eliminating the requirement for radiation exposure. The main drawback of using an ICE-guided method for CTI ablation is the additional cost associated with its implementation. However, when compared to the extra expenses incurred by utilizing EAMSs, this expenditure is found to be similar, (29) or potentially lower, particularly if the use of reprocessed ICE catheters is permitted (30).

## Limitations

Our study was conducted at a single center with a highly skilled operator in the use of ICE; therefore, our findings cannot be generalized. Additionally, the number of patients included in the study was limited. The relatively small sample size indeed brings about limitations in establishing conclusive results regarding both non-inferiority and equivalence. The reported acute success rates [100% (95% CI: 91%–100%)] and the lack of complications [0% (95% CI: 0%–8.8%)] are indicative, however, a larger sample size would be necessary to draw more definitive conclusions. Besides, according to the study protocol, the aim was to compare procedural data between the groups, and long-term follow-up data are not available. Further research with a more substantial sample could provide a clearer picture of the approach's effectiveness and safety*.* To gain a better understanding of potential advantages in clinical outcomes, multicenter trials are warranted. Finally, as previously discussed, it is essential to take into account the additional cost associated with utilizing an ICE catheter. Moreover, the use of ICE necessitates a larger venous sheath, but this did not result in an increase in access site complications.

## Conclusion

Our findings suggest that zero-fluoroscopy catheter ablation for typical AFL, guided exclusively by ICE, is both feasible and safe. Furthermore, we found that this technique does not significantly impact procedural data, safety, or efficacy when compared to standard ICE-guided CTI ablations. Further investigation is warranted for broader validation.

## Data Availability

The raw data supporting the conclusions of this article will be made available by the authors, without undue reservation.

## Supplementary material

Supplementary Video S1 Intracardiac echocardiography (ICE) visualization of the ablation catheter during cavotricuspid isthmus ablation.

## References

[1] Granada J Uribe W Chyou PH Maassen K Vierkant R Smith PN Incidence and predictors of atrial flutter in the general population. J Am Coll Cardiol. (2000) 36(7):2242–6. 10.1016/S0735-1097(00)00982-7 11127467

[2] Brugada J Katritsis DG Arbelo E Arribas F Bax JJ Blomstrom-Lundqvist C 2019 ESC guidelines for themanagement of patients with supraventricular tachycardia. Eur Heart J. (2020) 41(5):655–720. 10.1093/eurheartj/ehz467 31504425

[3] Giehm-Reese M Kronborg MB Lukac P Kristiansen SB Nielsen JM Johannessen A Recurrent atrial flutter ablation and incidence of atrial fibrillation ablation after first-time ablation for typical atrial flutter: a nation-wide danish cohort study. Int J Cardiol. (2020) 298:44–51. 10.1016/j.ijcard.2019.07.077 31521436

[4] Bencsik G Pap R Makai A Klausz G Chadaide S Traykov V Randomized trial of intracardiac echocardiography during cavotricuspid isthmus ablation. J Cardiovasc Electrophysiol. (2012) 23(9):996–1000. 10.1111/j.1540-8167.2012.02331.x 22812499

[5] Lehar F Szegedi N Hejc J Jez J Soucek F Kulik T Randomized comparison of atrioventricular node re-entry tachycardia and atrial flutter catheter ablation with and without fluoroscopic guidance: zerofluoro study. Europace. (2022) 24(10):1636–44. 10.1093/europace/euac049 35979596

[6] Debreceni D Janosi K Vamos M Komocsi A Simor T Kupo P. Zero and minimal fluoroscopic approaches during ablation of supraventricular tachycardias: a systematic review and meta-analysis. Front Cardiovasc Med. (2022) 9(April):1–10. 10.3389/fcvm.2022.856145 PMC9037593 35479287

[7] National Research Council. BEIR VII: health risks from exposure to low levels of ionizing radiation: report in brief. Natl Acad. (2006) 93:93–6. 10.17226/11340

[8] Yeung AWK. The “as low as reasonably achievable” (ALARA) principle: a brief historical overview and a bibliometric analysis of the most cited publications. Radioprotection. (2019) 54(2):103–9. 10.1051/radiopro/2019016

[9] Da Costa A Faure E Thévenin J Messier M Bernard S Abdel K Effect of isthmus anatomy and ablation catheter on radiofrequency catheter ablation of the cavotricuspid isthmus. Circulation. (2004) 110(9):1030–5. 10.1161/01.CIR.0000139845.40818.75 15326078

[10] Scaglione M Caponi D Di Donna P Riccardi R Bocchiardo M Azzaro G Typical atrial flutter ablation outcome: correlation with isthmus anatomy using intracardiac echo 3D reconstruction. Europace. (2004) 6(5):407–17. 10.1016/j.eupc.2004.05.008 15294265

[11] Casella M Dello Russo A Pelargonio G Del Greco M Zingarini G Piacenti M Near zerO fluoroscopic exposure during catheter ablation of supraventricular arrhythmias: the NO-PARTY multicentre randomized trial. Europace. (2016) 18(10):1565–72. 10.1093/europace/euv344 26559916 PMC5072134

[12] Regoli F Faletra F Marcon S Leo LA Dequarti MC Caputo ML Anatomic characterization of cavotricuspid isthmus by 3D transesophageal echocardiography in patients undergoing radiofrequency ablation of typical atrial flutter. Eur Heart J Cardiovasc Imaging. (2018) 19(1):84–91. 10.1093/ehjci/jew336 28180237

[13] Houmsse M Daoud EG. Radiation exposure: a silent complication of catheter ablation procedures. Heart Rhythm. (2012) 9:715–6. 10.1016/j.hrthm.2012.01.015 (Elsevier B.V) 22289168

[14] Orme NM Rihal CS Gulati R Holmes DR Lennon RJ Lewis BR Occupational health hazards of working in the interventional laboratory a multisite case control study of physicians and allied staff. (2015).

[15] MacÍas R Uribe I Tercedor L Jiménez J Barrio T Álvarez M. A zero-fluoroscopy approach to cavotricuspid isthmus catheter ablation: comparative analysis of two electroanatomical mapping systems. Pacing Clin Electrophysiol. (2014) 37(8):1029–37. 10.1111/pace.12376 24628051

[16] Ventura R Rostock T Klemm HU Lutomsky B Demir C Weiss C Catheter ablation of common-type atrial flutter guided by three-dimensional right atrial geometry reconstruction and catheter tracking using cutaneous patches: a randomized prospective study. J Cardiovasc Electrophysiol. (2004) 15(10):1157–61. 10.1046/j.1540-8167.2004.04064.x 15485440

[17] Schoene K Rolf S Schloma D John S Arya A Dinov B Ablation of typical atrial flutter using a non-fluoroscopic catheter tracking system vs. Conventional fluoroscopy—results from a prospective randomized study. Europace. (2015) 17(7):1117–21. 10.1093/europace/euu398 25736724

[18] Bulava A Hanis J Eisenberger M. Catheter ablation of atrial fibrillation using zero-fluoroscopy technique: a randomized trial. Pacing Clin Electrophysiol. (2015) 38(7):797–806. 10.1111/pace.12634 25790320

[19] Lurie A Amit G Divakaramenon S Acosta JG Healey JS Wong JA. Outcomes and safety of fluoroless catheter ablation for atrial fibrillation. CJC Open. (2021) 3(3):303–10. 10.1016/j.cjco.2020.11.002 33778447 PMC7984996

[20] Lyan E Tsyganov A Abdrahmanov A Morozov A Bakytzhanuly A Tursunbekov A Nonfluoroscopic catheter ablation of paroxysmal atrial fibrillation. Pacing Clin Electrophysiol. (2018) 41(6):611–9. 10.1111/pace.13321 29566268

[21] Percell J Sharpe E Percell R. SANS FLUORO (SAy No series to FLUOROscopy): a first-year experience. J Innov Cardiac Rhythm Manage. (2016) 7(11):2529–34. 10.19102/icrm.2016.071102

[22] Zei PC Quadros KK Clopton P Thosani A Ferguson J Brodt C Safety and efficacy of minimal- versus zerofluoroscopy radiofrequency catheter ablation for atrial fibrillation: a multicenter, prospective study. J Innov Cardiac Rhythm Manage. (2020) 11(11):4281–91. 10.19102/icrm.2020.111105 PMC7685314 33262896

[23] Debreceni D Janosi K Bocz B Turcsan M Lukacs R Simor T Zero fluoroscopy catheter ablation for atrial fibrillation: a systematic review and meta-analysis. Front Cardiovasc. (2023) 10:1178783. 10.3389/fcvm.2023.1178783 PMC10313423 37396578

[24] Janosi K Debreceni D Janosa B Simor T Kupo P. Visualizable vs. standard, non-visualizable steerable sheath for pulmonary vein isolation procedures: randomized, single-center trial. Front Cardiovasc Med. (2022) 24(Supplement_1):1–7. 10.3389/fcvm.2022.1033755 PMC9709402 36465461

[25] Kupo P Saghy L Bencsik G Kohari M Makai A Vamos M Randomized trial of intracardiac echocardiography-guided slow pathway ablation. J Interv Card Electrophysiol. (2022) 63(3):709–14. 10.1007/s10840-022-01126-y 35044581

[26] Goya M Frame D Gache L Ichishima Y Tayar DO Goldstein L The use of intracardiac echocardiography catheters in endocardial ablation of cardiac arrhythmia: meta-analysis of efficiency, effectiveness, and safety outcomes. J Cardiovasc Electrophysiol. (2020) 31(3):664–73. 10.1111/jce.14367 31976603 PMC7078927

[27] Herman D Osmancik P Zdarska J Prochazkova R. Routine use of intracardiac echocardiography for atrial flutter ablation is associated with reduced fluoroscopy time, but not with a reduction of radiofrequency energy delivery time. J Atr Fibrillation. (2017) 10(2):1553. 10.4022/jafib.1553 29250227 PMC5673286

[28] 28. Luani B Ismail A Kaese S Pankraz K Schmeisser A Wiemer M Zero-fluoroscopy ablation of the cavotricuspid isthmus guided by intracardiac echocardiography in patients with typical atrial flutter. ESC Congress. (2022).

[29] Winkle RA Mead RH Engel G Kong MH Patrawala RA. Physician-controlled costs: the choice of equipment used for atrial fibrillation ablation. J Interv Card Electrophysiol. (2013) 36(2):157–65. 10.1007/s10840-013-9782-x 23483336 PMC3606509

[30] Bank AJ Berry JM Wilson RF Lester BR. Acceptance criteria for reprocessed AcuNav® catheters: comparison between functionality testing and clinical image assessment. Ultrasound Med Biol. (2009) 35(3):507–14. 10.1016/j.ultrasmedbio.2008.09.013 19056163

