# Supplementary material for: Feasibility and safety of cavotricuspid isthmus ablation using exclusive intracardiac echocardiography guidance: a proof-of-concept, observational trial
Source: Front Cardiovasc Med. 2023 Oct 12;10:1244137. doi: 10.3389/fcvm.2023.1244137 (PMC10601457; doi:10.3389/fcvm.2023.1244137)
Supplement: Supplementary file 1 [file Table1.docx]

|  | **Standard ICE group (n=40)** | **Zero ICE group (n=40)** | **p value** |
| --- | --- | --- | --- |
| **Age (years)** | 49.6±14.9 | 53.0±13.4 | 0.15 |
| **Male (%)** | 28 (70) | 27 (67.5) | 0.81 |
| **Hypertension (%)** | 30 (75) | 35 (87.5) | 0.15 |
| **Diabetes mellitus (%)** | 14 (35) | 18 (45) | 0.36 |
| **Heart failure (%)** | 13 (32.5) | 8 (20) | 0.20 |
| **Coronary artery disease (%)** | 9 (22.5) | 12 (30) | 0.44 |
| **Chronic kidney disease (%)** | 0 (0) | 3 (7.5) | NA |
| **Prior stroke/TIA (%)** | 6 (15) | 3 (7.5) | 0.32 |
| **Atrial fibrillation (%)** | 5 (12.5) | 10 (25) | 0.15 |
| **COPD (%)** | 2 (5) | 1 (2.5) | 0.56 |
| **LA diameter (mm)** | 59.0 ± 9.0 | 58.9 ± 7.9 | NA |
| **Ongoing typical AFL (%)** | 23 (57.5) | 21 (52.5) | 0.65 |

**Clinical characteristics of the study population.** Abbreviations: AFL: atrial flutter; COPD: chronic obstructive pulmonary disease; ICE: intracardiac echocardiography; LA: left atrium, NA: not available; TIA: transient ischemic attack
