# Supplementary material for: Feasibility and safety of cavotricuspid isthmus ablation using exclusive intracardiac echocardiography guidance: a proof-of-concept, observational trial
Source: Front Cardiovasc Med. 2023 Oct 12;10:1244137. doi: 10.3389/fcvm.2023.1244137 (PMC10601457; doi:10.3389/fcvm.2023.1244137)
Supplement: Supplementary file 2 [file Table2.docx]

|  | **Standard ICE group (n=40)** | **Zero ICE group (n=40)** | **p value** |
| --- | --- | --- | --- |
| **Total procedure time (min)** | 55.5 (46.5; 66.8) | 51.5 (44.0; 65.5) | 0.50 |
| **From puncture to first ablation (min)** | 18 (13.5; 23) | 19 (15; 23.5) | 0.50 |
| **Total ablation time (sec)** | 597 (447; 908) | 430 (260; 750) | 0.02 |
| **Total ablation energy (Ws)** | 22458 (14836; 31116) | 17043 (10533; 29302) | 0.10 |
| **From first to last ablation time (min)** | 16 (10; 31) | 12 (5; 25.5) | 0.16 |
| **From puncture to last ablation time (min)** | 35 (27; 50) | 32 (24; 46.5) | 0.51 |
| **Total fluoroscopic time (sec)** | 57 (36.3; 90)  range: 29 - 184 | 0 (0; 0)  range: 0 - 78 | <0.001 |
| **Total fluoroscopy dose (mGy)** | 3.17 (2.27; 5.63) | 0 (0; 0) | <0.001 |
| **First pass block (%)** | 55.0 | 55.0 | 1.0 |
| **Acute reconnection (%)** | 25.3 | 35.0 | 0.31 |
| **Acute success (%)** | 100 | 100 | 1.0 |
| **Complication (%)** | 0 | 0 | NA |

**Table 2. Procedural parameters in the study population.** Abbreviations: ICE: intracardiac echocardiography; NA: not available;
